# Dual-Task Performance in Individuals With Chronic Obstructive Pulmonary Disease: A Systematic Review With Meta-Analysis

**DOI:** 10.1155/2024/1230287

**Published:** 2024-08-10

**Authors:** Joselyn González Pasten, Jennifer Campos Aguayo, Javiera Aburto, Felipe Araya-Quintanilla, Alejandro Álvarez-Bustos, Juan José Valenzuela-Fuenzalida, Pat G. Camp, Walter Sepúlveda-Loyola

**Affiliations:** ^1^ Facultad de Salud y Ciencias Sociales Universidad de Las Américas, Santiago, Chile; ^2^ Grupo de Estudiantes de Iniciación Científica en Kinesiología (GICK) Universidad de Las Américas, Santiago, Chile; ^3^ Escuela de Kinesiología Facultad de Odontología y Ciencias de la Rehabilitación Universidad San Sebastián, Santiago, Chile; ^4^ Biomedical Research Center Network for Frailty and Healthy Ageing (CIBERFES) Institute of Health Carlos III, Madrid, Spain; ^5^ Departamento de Morfología Facultad de Medicina Universidad Andrés Bello, Santiago 8370186, Chile; ^6^ Department of Physical Therapy University of British Columbia, Vancouver, British Columbia, Canada

**Keywords:** chronic obstructive pulmonary disease, dual tasks, older adults, physical assessment, physical performance

## Abstract

**Background:** Chronic obstructive pulmonary disease (COPD) is characterized by important extrapulmonary alterations that could affect the performance in dual task (DT) (motor and cognitive tasks executed simultaneously), which is defined as DT interference (DTI).

**Objective:** To compare the performance of DT between individuals with COPD and healthy control subjects (HCSs).

**Methods:** The literature search was conducted in seven databases (Medline, Scopus, Web of Science, PEDro, SciELO, LILACS, and Google Scholar) up to December 2023, including studies published in English, Spanish, or Portuguese. Studies with individuals diagnosed with COPD older than 60 years, who were evaluated with any DT assessment, and compared with HCS were included. The quality of the studies was evaluated using the risk of bias in nonrandomized studies of interventions (ROBINS-I). The meta-analysis was performed with JAMOVI software 5.4. The study protocol was registered on PROSPERO (CRD42023435212).

**Results:** From a total of 128 articles, 5 observational studies were selected in this review, involving 252 individuals aged between 60 and 80 years, from France, Italy, Canada, Turkey, and Belgium. Notable DTI was observed in individuals with COPD compared to HCS (standard mean difference [SMD] = 0.91; 95% confidence interval (CI) 0.06–1.75, *p* = 0.04). Individuals with COPD had impaired gait speed, balance control, muscle strength, and cognitive interference during DT compared to HCS. DT assessment protocols included different combination of motor and cognitive tasks, using functional test, gait analysis, and muscle strength paired with countdown and verbal fluency tasks. Studies presented low (*n* = 2), moderate (*n* = 1), and serious (*n* = 2) overall risk of bias.

**Conclusion:** Older adults diagnosed with COPD exhibited a significant DTI compared to HCSs, which is characterized by poorer physical and cognitive performance during DT execution. These findings highlight the importance of incorporating DT assessments into clinical practice for individuals with COPD.

## 1. Introduction

Chronic obstructive pulmonary disease (COPD) is a preventable and treatable disease, characterized by persistent respiratory symptoms and airflow limitation resulting from the inhalation of toxic gases [1] primarily associated with tobacco consumption [2]. COPD is characterized by several extrapulmonary impairments, including muscular weakness, decreased aerobic capacity, postural balance disorders, and an increased risk of falls [3]. These physical impairments in individuals with COPD negatively diminish the performance in activities of daily life [4] and quality of life, often leading to symptoms of depression and anxiety [5]. Moreover, these patients also present cognitive impairment in terms of attention, memory, and concentration deficits [6]. The interaction of these physical and cognitive consequences in COPD older adults may impede the ability to perform daily activities and maintain an independent lifestyle [7].

Dual-task (DT) performance is defined as the simultaneous execution of a cognitive task and a motor task [8]. The evaluation of DT is closely associated with the functionality of older adults, since during the performance of daily activities, multiple motor and cognitive tasks are executed simultaneously [2]. DT performance can be evaluated by incorporating additional cognitive tests such as working memory, verbal fluency, or arithmetic tasks alongside motor tasks such as walking, static and dynamic balance, and muscle strength assessments [9]. Notably, assessing motor and cognitive tasks simultaneously can reveal both physical and cognitive impairments, elucidating the phenomenon known as DT interference (DTI) [10]. Studies have indicated that individuals with COPD present high DTI, exhibiting reductions in gait speed, postural balance control, and muscle strength when a cognitive task is added to the motor task [11]. The performance in DT has been linked to key clinical variables such as dementia, sarcopenia, and frailty [12], which are comorbidities associated with worse prognosis in individuals with COPD [13]. Additionally, current studies have reported that individuals with COPD may exhibit lower performance in single tasks and DTs compared to those without this respiratory disease [14–16]. Therefore, the assessment of DT in patients with COPD is crucial for analyzing the cognitive and motor impact of this respiratory condition, which can have significant implications for their functional capacity and comprehensive clinical management [2]. However, based on our knowledge, there are no previous systematic reviews that have compared the performance of DTs in patients with COPD and healthy subjects. Therefore, the aim of this systematic review is to compare the performance in DT performance in older adults with COPD and healthy control subjects (HCSs). In addition, our secondary objective is to identify primary assessment protocols for DTs (both motor and cognitive) in individuals with COPD.

## 2. Methods

We performed this review by following the Meta-analysis of Observational Studies in Epidemiology (MOOSE) [17] statement and the recommendations of the *Cochrane Collaboration Handbook* [18]. This review was reported and summarized using the Preferred Reporting Items for Systematic Reviews and Meta-Analysis (PRISMA) recommendations [19]. The protocol for this systematic review was registered in PROSPERO (CRD42023435212).

### 2.1. Search Strategy and Inclusion Criteria

The systematic search was conducted across seven databases (Medline, SciELO, Scopus, Web of Science, PEDro, LILACS, and Google Scholar) until May 2024, using a combination of the following Medical Subject Headings (MeSH) terms: COPD, COAD, Pulmonary Disease, and Chronic Obstructive with the free-text terms dual task, motor task, cognitive task, and multitask (Table S1). Additionally, a manual search of the reference lists of each included article was performed to identify potential additional studies. Studies that met the following criteria were considered: (1) population: individuals of any age range, diagnosed with COPD as defined by GOLD (Global Initiative for Chronic Obstructive Lung Disease) [20]; (2) exposure: individuals evaluated with DT assessments defined as the simultaneous execution of one cognitive and one motor tasks [8]; (3) comparator: individuals with COPD compared to age- and sex-matched HCSs; and (4) outcome: DT performance (motor and/or cognitive dual task interference) and protocols used to asses DT. We included observational studies published in English, Spanish, or Portuguese. Manuscripts in which DT assessment protocol was not detailed and/or included participants with decompensated COPD were excluded [8].

### 2.2. Data Extraction

Two independent reviewers (J.G.P. and J.C.A.) assessed the eligibility of articles based on title and abstract, categorizing citations as “include,” “exclude,” or “maybe.” Those deemed as “include” or “maybe” were further reviewed in full text. Any disagreements were resolved through consultation with a third independent advisor (W.S.L.). The total number of studies was stored in a single file. Results from the database searches were cross-checked using reference management software (Mendeley Reference Manager) and START (State of the Art through Systematic Review) [21], and duplicates were excluded. Data extraction was performed by two team members using standardized templates adapted to meet the study's objectives.

### 2.3. Risk of Bias Assessment

Risk of bias was appraised using ROBINS-I (risk of bias in nonrandomized studies of interventions) [22]. This tool assesses the risk of bias according to seven domains: confounding bias, participant selection bias, intervention classification bias, deviation from intended interventions bias, missing data bias, outcome measurement bias, and reporting bias. Each domain can be classified as having a “low,” “moderate,” or “serious” risk of bias. Data extraction and study assessment were conducted independently by two reviewers (F.A.Q. and J.G.P.). For any discrepancies, a third reviewer (W.S.L.) was included to reach a consensus.

### 2.4. Statistical Analysis

The Hartung–Knapp–Sidik–Jonkman random effect model was used with the Knapp and Hartung adjustment, for the comparison of DT performance using DTI between COPD and healthy controls. We performed the meta-analysis to compute a pooled estimate using standard mean difference (SMD) and respective 95% confidence intervals (CIs) for total outcomes between trials [23]. We compared the DTI in motor task between COPD and healthy controls, analyzing the interference of cognitive task on the motor performance assessed with functional test, balance test, walking test, and muscle strength assessments. The heterogeneity of results across studies was evaluated using the visual inspection (overlapping CI) and *I*^2^ statistic, which considers 0%–40% as may not be important, 30%–60% as moderate heterogeneity, 50%–90% as substantial heterogeneity, and 75%–100% as considerable heterogeneity [24]. The corresponding *p* values (*p* < 0.05) were also considered. The meta-analysis was performed with JAMOVI software 5.4 [25]. Additionally, publication bias was evaluated through a visual inspection of funnel plots and using the method proposed by Sterne, Egger, and Smith [26].

### 2.5. Grading of Recommendation, Assessment, Development, and Evaluation (GRADE)

The GRADE approach was used to assess the overall certainty of evidence [27]. The GRADE included the assessment of study limitations, consistency of effect, indirectness, imprecision, and publication bias. The quality of the evidence was classified into four categories: high, moderate, low, and very low. We used the GRADE profiler (GRADEpro) to create a “summary of findings.”

## 3. Results

### 3.1. Study Characteristics

**Figure 1:**
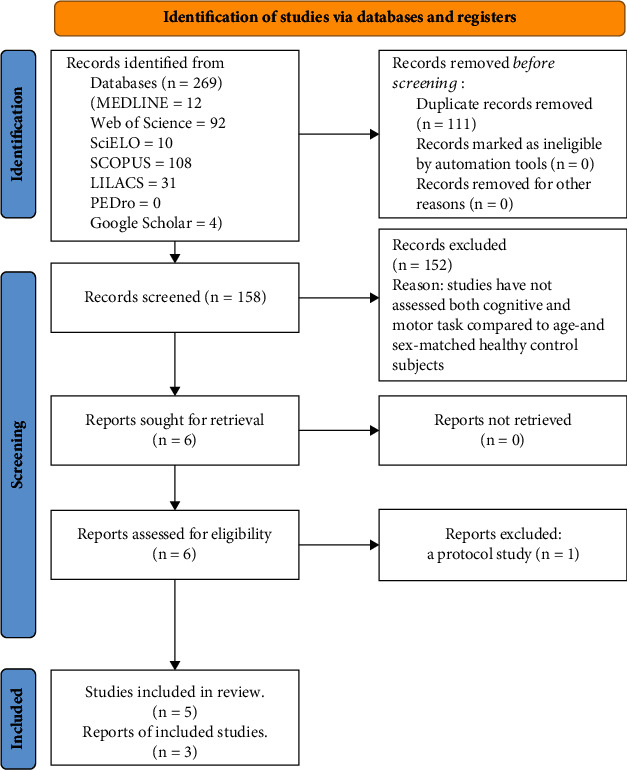
PRISMA flow diagram of article selection.

Of the 269 articles identified in our initial search, 5 observational studies evaluating DTs in individuals with COPD were selected (Figure 1 presents the details of the search and study selection process). Characteristics of the studies included are displayed in Table 1. The studies were performed in France [15], Italy [16], Canada [11], Turkey [12], and Belgium [28] involving a total of 252 individuals (COPD: 132 and HCS: 120), aged from 60 to 80 years with 70% being male. The I, II, III, and IV GOLD stage was 8%, 51%, 13%, and 28%, respectively [12, 16]. Since individuals with COPD were compared to age- and sex-matched HCSs, nondifferences were observed in age and sex between groups.

### 3.2. DT Assessment Protocols

To evaluate motor performance, gait analysis, muscle force production, and postural control were administered as motor tasks combined with different cognitive tasks. Gait analysis was measured using the functional test Timed Up and Go (TUG) [12, 16] and using movement analysis with Zeno walkway (ProtoKinetics Movement Analysis Software) [11] and Locomètre (Locomètre®–Satel, Blagnac, France) [15]. Gait analysis was measured from 3 to 15 m in a normal speed [11, 15]. Muscle force production (knee extension muscle strength) was assessed using the Biodex System 4-Pro Isokinetic Strength Dynamometer® [12]. Postural control with eyes open and with eyes closed was analyzed using Wii Balance Board [28] (Table 1).

To evaluate cognitive performance, calculation and verbal fluency tasks were used. For the calculation task, individuals with COPD were instructed to count backward in three, commencing from a randomly chosen number between 100 and 130 [11, 12, 15, 16, 28]. As part of the verbal fluency task, individuals with COPD were required to spell five-letter words in reverse [11] or to name as many animals as possible [28]. As an indicator of cognitive performance, the rate of correct responses per second was measured [12, 15]. Finally, to analyze the DT performance, DTI was calculated using formulas [11, 12] (Table 2).

### 3.3. DT Performance

**Figure 2:**
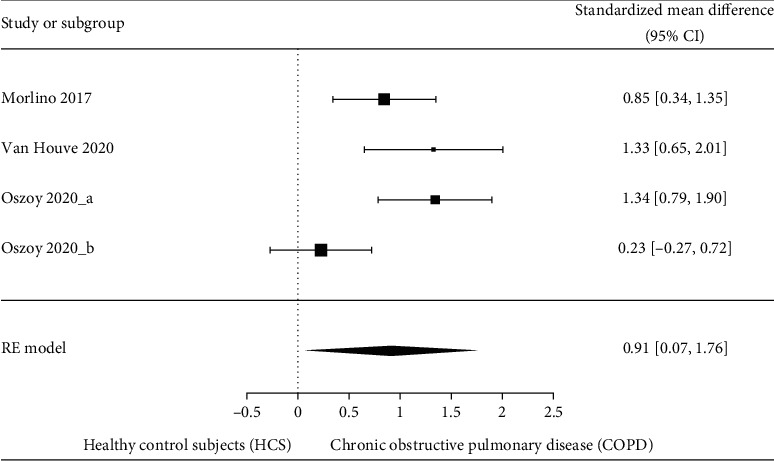
Forest plot of the comparison of dual-task interference between individuals with COPD and HCS. Heterogeneity: Tau = 0.45, chi^2^ = 3.549, df = 3 (*p* = 0.012), and *I*^2^ = 71.82%. Test for overall effect: *Z* = 3.37 (*p* < 0.001).

**Figure 3:**
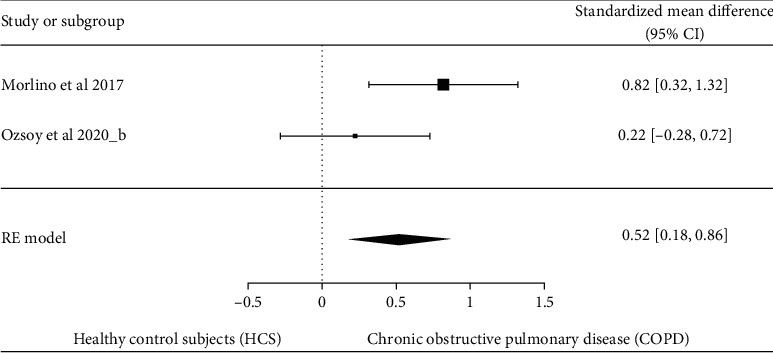
Forest plot of the comparison of dual-task interference between individuals with COPD and HCS (including only performance in TUG). Heterogeneity: Tau = 0.232, chi^2^ = 5.458, df = 3 (*p* = 0.003), and *I*^2^ = 45.04%. Test for overall effect: *Z* = 3.013 (*p* = 0.0026).

The comparison of DT performance between individuals with COPD and HCS is presented in Figure 2. Individuals with COPD showed higher DTI compared to HCS (SMD = 0.91 [95% CI 0.06–1.75], *p* = 0.04), with substantial heterogeneity (*I*^2^ 71.8%; *p* = 0.012). Since the study of I. Ozsoy et al. [12] analyzed the DTI in muscle force production and gait speed, we included both variables in the meta-analysis (identified with the letter “a” and “b” in Figure 2, respectively). Additionally, Figure 3 presents a separate analysis including only the DTI during TUG using SMD since TUG performance was presented as total time [16] and as gait speed [12] (SMD = 0.52 [95% CI 0.18–0.86], *p* = 0.003), with moderate heterogeneity (*I*^2^ 45%; *p* = 0.14). Individuals with COPD exhibited a higher DTI characterized by a decrease in gait speed [11, 15, 16], heightened variability of step length [15, 16], increased balance control perturbation [28], diminished muscle force production [12], and higher cognitive interference [11, 12] during DT compared to HCS (Table 1). There was a moderate quality of evidence according to the GRADE rating (Table 3).

### 3.4. Publication Bias and Risk of Bias

The publication bias was evaluated through a visual inspection of funnel plots and using the Egger regression showing no statistically significant differences for the publication bias (Egger = 1.4, *p* = 0.280) (Figure S1). The scale used was the standardized mean difference.  Table 4 presents the results of the risk of bias conducted using the ROBINS-I tool. Two studies [11, 12] (40%) reported a moderately low risk of bias. Another two studies [15, 16] (40%) reported a serious risk of bias. Finally, only one study (20%) was categorized as having a moderate risk of bias [11].

## 4. Discussion

The main finding of this systematic review with meta-analysis is that individuals with COPD encounter significant difficulties in executing DTs simultaneously, resulting in high DTI characterized by poorer performance in motor and cognitive tests compared with their counterpart HCS [11, 14–16, 29]. These difficulties may be manifested in a significant reduction in terms of gait speed [11, 15, 16], perturbation in balance control [28], diminished muscle force production [12] and worse cognitive performance [11, 12].

The proper status of both physical and cognitive function is essential for the performance of daily activities, in which the simultaneous execution of multiple cognitive and motor tasks interacts, such as walking while talking, thinking, or avoiding obstacles [10]. In this context, when cognitive and motor tasks were executed simultaneously, individuals with COPD presented a high DTI, causing a decrease in the performance of one or both tasks [11, 14–16, 29]. The impairment of both physical and cognitive function separately is well documented in individuals with COPD [30–33], potentially restricting the execution of DT activities, influencing their daily activity performance and overall quality of life [11, 12].

The observed DTI in individuals with COPD can be attributed to several interrelated factors. First, individuals with COPD presented lower gait speed, muscle weakness, higher risk of falls, and more risk of sarcopenia and frailty compared to HCS [33]. In this sense, this decline in motor task seems to become more pronounced when a cognitive task is introduced [11, 14–16, 29]. Morlino et al. observed a reduced gait speed, variability of step length, and longer duration of TUG test in COPD patients compared to HCS [16]. Furthermore, these gait changes were more pronounced when the cognitive task was added to TUG test [16]. The reduction in gait speed is a potential compensatory strategy developed to decrease the complexity of the task being performed to maintain postural stability and postural balance [11]. In addition, reduced gait speed is associated with increased risk of falls and disability [33]. This reduction in physical performance can be explained by the cognitive–motor interference, since it has been reported that walking with simultaneous performance of a cognitive task increased the cognitive demands in this population [34].

Cognitive impairment is another well-known consequence in patients with COPD [35]. Chronic hypoxia due to airway obstruction may negatively impact brain function, including attention, memory, and concentration in individuals with this respiratory disease [34]. Moreover, sleep apnea, a common comorbidity in COPD patients, can exacerbate hypoxia during sleep, further affecting cognitive function and potentially impacting the ability to perform DTs [36]. Regarding brain function, studies have demonstrated that oxygenated hemoglobin, which influences the oxygen transport capacity in the blood, is linked to the production of brain-derived neurotrophic factor (BDNF), a crucial protein for brain health [37]. Changes in BDNF levels can impact in memory, learning, and other cognitive functions, potentially affecting the ability to perform DTs. In this context, a study by Hassan et al. [11] analyzed the cognitive and motor performance during DTs and related dorsolateral prefrontal cortex changes in oxygenated hemoglobin in individuals with COPD. This study demonstrated that the activation of the left and right dorsolateral prefrontal cortex were higher during single-task walking in patients with COPD compared to HCS. However, these levels did not exhibit further increase during DT execution in patients with this disease. In contrast, there was an observed increasement in oxygenated hemoglobin during DT in HCS [34]. The lack of further increase in oxygenated hemoglobin from single to DT in patients with COPD may indicate a diminished cognitive–motor capacity, which can interfere in locomotor and static activities in these individuals.

This systematic review highlights the importance of considering both cognitive and motor function when assessing the functional capacity in COPD (11). Most of the protocols to assess DT in individuals with COPD are highly applicable in routine clinical practice due to the low cost and time efficiency involved in administering these tests [11, 12, 15, 16, 28]. The primary protocol for assessing motor task in COPD patients is the gait speed test calculated from 3 to 15 m assessed at a usual pace [11, 15]. Counting backward in 3, commencing from a randomly chosen number between 100 and 130 was the most utilized cognitive task [11, 12, 15, 16, 28]. Both tests could be easily conducted in the clinical setting. Finally, calculating DTI using mathematical formulas [11, 12] is recommended to analyze the DT performance (Table 2), although normative values are not available. Assessing the ability to perform DT in this population is crucial for understanding the interaction of COPD with cognitive and motor aspects, holding relevant implications for their comprehensive medical care [14]. Despite the growing awareness of the impact of COPD on DT performance, there are only five studies published in this field, the evaluation of these tasks is infrequent in clinical practice, and there is a lack of exercise intervention with DT in this population [38]. Therefore, more studies are necessary in this field. In addition, since COPD is highly associated with several geriatric entities such as sarcopenia [33] or frailty [4]; these entities should be considered in future studies since DT performance seems to be reduced when these entities are present [39, 40]. Consequently, it is crucial for future research to focus on developing specific therapeutic interventions to enhance DT performance in patients with COPD, considering the benefits of DT training on static and dynamic postural stability in older adults [41]. This approach is paramount for addressing consequences beyond the pulmonary aspects of COPD, with the aim of improving the quality of life for patients and alleviating the economic burden associated with this disease.

Our results should be considered with caution due to the following limitations. First, the variability in the assessment protocols used to assess DT. Second, the studies have shown high and moderate risk of bias. Third, due to the small number of studies included in the qualitative synthesis and high heterogeneity, the accuracy of the meta-analysis may be affected.

## 5. Conclusion

Individuals with COPD exhibit an augmented DTI during DT compared to HCSs, characterized by reduction in gait speed, balance, muscle strength and cognitive performance. Protocols to assess DT primarily combined walking activities with a cognitive task, such as counting backward or verbal fluency. These findings contribute valuable insights into the impact of COPD on DT performance and highlight the need for standardized assessment and treatment protocols.

## Figures and Tables

**Table 1:** Characteristics of the studies included in the systematic review.

**Author and year**	**Country**	**Study design**	**Sample size (** **n** **) (total, COPD, and HCS)**	**Male (%) (COPD and HCS)**	**Age (years) (COPD and HCS)**	**GOLD stage (I/II/III/IV) (** **n** **)**	**Dual-task assessment**		**Main results**
							**Motor task**	**Cognitive task**	
I. Ozsoy et al., 2021 [12]	Turkey	Cross-sectional controlled study	Total: 62 COPD: 35 HCS: 27	COPD: 89% HCS: 84% ^∗^No statistical differences	COPD: 62.13 ± 8.17 HCS: 60.6 ± 7.84 ^∗^No statistical differences	3/23/7/2	Motor Task 1: The participants were asked to perform the isokinetic muscle test (knee extension muscle strength) Motor Task 2: TUG test was used to assess functional balance and mobility. The participants were asked to rise from a chair, walk 3 m, turn around, walk back to the chair, and sit down	Counting down in intervals of 3, starting from 100	The deterioration in cognitive performance during dual task (i.e., muscle force production together with a cognitive task) was more pronounced in patients with COPD than in HCS ^∗^No difference was observed during the TUG
Morlino et al., 2017 [16]	Italy	Cross-sectional study	Total: 68 COPD: 40 HCS: 28	COPD: 73% HCS: 57% ^∗^No statistical differences	COPD: 70.7 ± 7.1 HCS: 69.5 ± 6.7 ^∗^No statistical differences	3/15/3/19	TUG was used to assess functional balance and mobility. The participants were asked to rise from a chair, walk 3 m, turn around, walk back to the chair, and sit down	Counting down in intervals of 3	Balance and gait are affected in patients with COPD Gait changes were represented not only by reduced gait speed but also by increased variability of step length and longer duration of TUG test with dual task in patients than HCS
Heraud et al., 2018 [15]	France	Cross-sectional controlled study	Total: 45 COPD: 25 HCS: 20	No reported	COPD: 64.6 ± 7.4 HCS: 69.5 ± 6.7 ^∗^No statistical differences	No reported	The subjects were asked to walk at their own pace for 15 m. The task was performed in a quiet space along a flat corridor. No encouragement was given during the test	Counting down in intervals of 3 from 100	The arithmetic task during walking deteriorated the neural control of walking in the COPD patients Individuals with COPD exhibited an exaggerated cognitive interference during dual-task walking, characterized by an increase in gait variability, a parameter closely related to fall risk and fall history
Hassan et al., 2020 [11]	Canada	Cross-sectional study	Total: 35 COPD: 15 HCS: 20	COPD: 60% HCS: 46% ^∗^No statistical differences	COPD: 70.7 ± 8.0 HCS: 69.1 ± 6.9 ^∗^No statistical differences	No reported	The subjects were asked to walk at their own pace for 30 m. The task was performed in a quiet space along a flat corridor. No encouragement was given during the test	Cognitive Task 1: Participants spelled 5-letter words backwards from a list of 100 words for 1 min Cognitive Task 2: Counting down in intervals of 3 from 100	Patients with COPD walked slower than healthy adults and tended to have lower accuracy in the performance of the cognitive task
Van Hove et al., 2021 [28]	Belgium	Cross-sectional study	Total: 42 COPD: 21 HCS: 21	COPD: 67% HCS: 67% ^∗^No statistical differences	COPD: 64 ± 8 HCS: 69.5 ± 6.7 ^∗^No statistical differences	No reported	Postural control was assessed with eyes open and with eyes closed, during a quiet period of 60 s	Cognitive Task 1: A calculation task (“numbers”), during which the subjects had to count back in threes, starting from a number randomly selected between 120 and 130 Cognitive Task 2: A verbal fluency task (“words”), during which the participants had to name as many animals as possible	The addition of cognitive tasks while standing increased balance control perturbation and, thus, risk of falling in COPD patients, compared HCS

Abbreviations: COPD: chronic obstructive pulmonary disease; HCS: healthy control subject; TUG: Timed Up and Go test.

^∗^No statistical differences.

**Table 2:** Formulas used to analyze the dual-task performance.

**Dual-task interference (DTI)**	**Rate of correct responses per second (RCR)**
DTI motor (%) = (dual‐task TUG time − single − task TUG time)/single − task TUG time∗100	RCR = (correct answers/total time)∗(correct answers/total answers)
DTI cognitive (%) = (dual‐task RCR − single − task RCR)/single − task RCR∗100	

Abbreviations: DTI: dual-task interference; TUG: Timed Up and Go test; RCR: rate of correct responses per second.

**Table 3:** Summary of findings (SoF) and quality of evidence (GRADE) for dual-task in COPD patients and healthy subjects.

**Certainty assessment**								**No. of patients**		**Effect**		**Quality of evidence (GRADE)**	**Importance**
**No. of studies**	**Study design**	**Risk of bias**	**Inconsistency**	**Indirectness**	**Imprecision**	**Publication bias**	**Confounding factors**	**COPD**	**Healthy subjects**	**Relative (95% CI)**	**SMD (95% CI)**		
Performance dual task (overall)													
4	Observational	Not serious	Serious	Not serious	Serious	Not detected	Not controlled^∗^	96	75	—	SMD = 0.91 (0.06–1.75)	⨁⨁⨁◯ moderate	Important

*Note:* Quality of evidence. High: The research provides a very good indication of the likely effect. The probability that the effect is different is low. Moderate: The research provides a good indication of the likely effect. The probability that the effect is substantially different is moderate. Low: The research gives some indication of the probable effect. However, the probability that the effect is substantially different is high. Very low: The research does not provide a reliable estimate of the probable effect. The probability that the effect is substantially different is very high. Downgrading: GRADE approach has four reasons for possible rate down the quality of evidence. It begins with the study designs (trials or observational studies) and downgrading the evidence to two levels: (1) for study limitation if the majority of studies (> 50%) was rated as high risk of bias and (2) for inconsistency if heterogeneity was greater than the accepted low level *I*^2^ > 40%. Point estimates vary widely across studies. Confidence intervals (CIs) show minimal or no overlap. The statistical test for heterogeneity which tests the null hypothesis that all studies in a meta-analysis have the same underlying magnitude of effect shows a low *p* value. The *I*^2^ which quantifies the proportion of the variation in point estimates because the among-study difference is large. (3) For indirectness, directness was undoubled. (4) For imprecision, if meta-analysis had a small sample size (*n* < 400) or confidence interval very wide.

Abbreviations: CI: confidence interval, SMD: standard mean difference.

^∗^Any possible residual confounding factors could reduce the demonstrated effect.

**Table 4:** Risk of bias for each item for observational studies analyzed with ROBINS-I.

**Studies**	**D1**	**D2**	**D3**	**D4**	**D5**	**D6**	**D7**	**Overall risk**
Hassan 2020 [11]	Low	Low	Moderate	Low	Low	Serious	Low	Moderate
Heraud 2018 [15]	Moderate	Low	Moderate	Moderate	Low	Low	Serious	Serious
Morlino 2017 [16]	Moderate	Low	Moderate	Moderate	Low	Low	Serious	Serious
Ozsoy 2021 [12]	Low	Low	Moderate	Low	Low	Low	Serious	Low
Van Hove 2021 [28]	Low	Low	Moderate	Low	Low	Low	Serious	Low

*Note:* Risk of bias domains: D1, bias due to confounding; D2, bias in selection of participants into the study; D3, bias in classification of interventions; D4, bias due to deviations from intended interventions; D5, bias due to missing data; D6, bias in measurement of outcomes; D7, bias in selection of the reported result.

## Abbreviations

COPD: chronic obstructive pulmonary disease
DT: dual task
DTI: dual-task interference
GRADE: Grading of Recommendation, Assessment, Development, and Evaluation
HCS: healthy control subjects
MOOSE: Meta-analysis of Observational Studies in Epidemiology
RCR: rate of correct responses per second
ROBINS-I: risk of bias in nonrandomized studies of interventions
TUG: Timed Up and Go test

## Supporting Information

Supporting Information 1 Table S1: the search strategy used in each database until May 2024 (Medline, SciELO, Scopus, Web of Science, PEDro, LILACS, and Google Scholar) was reported.

Supporting Information 2 Figure S1: a funnel plot. In this figure, each study meta-analyzed was represented as a point. The vertical axis represents a measure of study precision using standard error. The *x*-axis displays the study estimated effect size for an outcome.
