# Supplementary material for: Dual-Task Performance in Individuals With Chronic Obstructive Pulmonary Disease: A Systematic Review With Meta-Analysis
Source: Pulm Med. 2024 Aug 10;2024:1230287. doi: 10.1155/2024/1230287 (PMC11330333; doi:10.1155/2024/1230287)
Supplement: Supporting Information 1 — Table S1: the search strategy used in each database until May 2024 (Medline, SciELO, Scopus, Web of Science, PEDro, LILACS, and Google Scholar) was reported. [file 1230287.f1.docx]

| Database | Search strategy | Results  May/ 07/ 2024 |
| --- | --- | --- |
| MEDLINE /PubMed | (((Pulmonary Disease, Chronic Obstructive[MeSH Terms]) OR (COPD[MeSH Terms])) OR (COAD[MeSH Terms])) AND ((((dual task[Title/Abstract]) OR (motor task[Title/Abstract])) OR (cognitive task[Title/Abstract])) OR (multitask task[Title/Abstract])) | 12 |
| Web of Science | Pulmonary Disease, Chronic Obstructive (All Fields) or COPD (All Fields) or coad (All Fields) AND dual task (All Fields) or motor task (All Fields) or cognitive task (All Fields) or multitask task (All Fields) | 92 |
| SCIELO | Expresión: ((pulmonary disease, chronic obstructive) OR (copd) OR (coad) OR (chronic obstructive pulmonary disease) OR (EPOC) OR (DPOC)) AND ((dual task) OR (Motor task) OR (cognitive task) OR (multi task) OR (motor) OR (cognitive) OR (task) OR (tarea dual) OR (dupla tarefa))  Study type: Article | 10 |
| SCOPUS | ( ( TITLE-ABS-KEY ( pulmonary AND disease, AND chronic AND obstructive ) OR TITLE-ABS-KEY ( copd ) OR TITLE-ABS-KEY ( coad ) ) ) AND ( ( TITLE-ABS-KEY ( dual AND task ) OR TITLE-ABS-KEY ( motor AND task ) OR TITLE-ABS-KEY ( cognitive AND task ) OR TITLE-ABS-KEY ( multitask AND task ) ) ) | 108 |
| PEDro | Abstract & Title: copd; Pulmonary Disease, Chronic Obstructive; COAD; dual task; motor task; cognitive task; multitask  Topic: no appropriate value in this field  Problem: no appropriate value in this field  Topic: no appropriate value in this field  Method: Clinical trial | 0 |
| LILACS | ab: (“pulmonary disease, chronic obstructive” OR copd OR coad OR “chronic obstructive pulmonary disease”) AND (“dual task” OR “motor task” OR “cognitive task” OR “multi task”) | 31 |
| Google scholar | allintitle: chronic obstructive pulmonary disease + dual task  allintitle: COPD + dual task  allintitle:Pulmonary Disease, Chronic Obstructive + dual task  allintitle:Pulmonary Disease, Chronic Obstructive + dual task  allintitle: pulmonary disease chronic obstructive pulmonary dual tarefa obstructive OR copd OR coad OR chronic OR disease OR EPOC OR DPOC OR task OR Motor OR task OR cognitive OR task OR multi OR task OR motor OR cognitive OR task OR tarea OR dual OR dupla | 4 |
| Total | | 269 |

**Table S1: Search Strategy**
