# Supplementary material for: Dual-Task Performance in Individuals With Chronic Obstructive Pulmonary Disease: A Systematic Review With Meta-Analysis
Source: Pulm Med. 2024 Aug 10;2024:1230287. doi: 10.1155/2024/1230287 (PMC11330333; doi:10.1155/2024/1230287)
Supplement: Supporting Information 2 — Figure S1: a funnel plot. In this figure, each study meta-analyzed was represented as a point. The vertical axis represents a measure of study precision using standard error. The x-axis displays the study estimated effect size for an outcome. [file 1230287.f2.docx]

**Figure 1S:** Funnel plot for publication bias


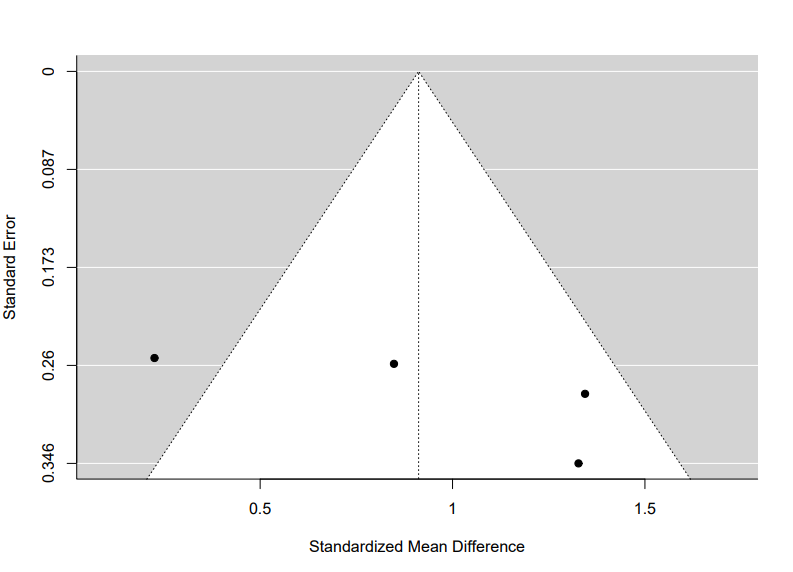


Description: Each study meta-analyzed was represented as a point. The vertical axis represents a measure of study precision using standard error. Larger studies with greater precision are displayed at the top and studies with lower precision at the bottom. The x-axis displays the study estimated effect size for an outcome. On the horizontal axis, the graph displays the estimated effect size of each study's outcome. The scale for was standardized mean difference.
